# Supplementary material for: Multicentric tracking of multiple agents by anterior cingulate cortex during pursuit and evasion
Source: Nat Commun. 2021 Mar 31;12:1985. doi: 10.1038/s41467-021-22195-z (PMC8012621; doi:10.1038/s41467-021-22195-z)
Supplement: Supplementary file 1 — Supplementary Information [file 41467_2021_22195_MOESM1_ESM.pdf]

Supplementary Material

Figure Caption for Supplementary Figures

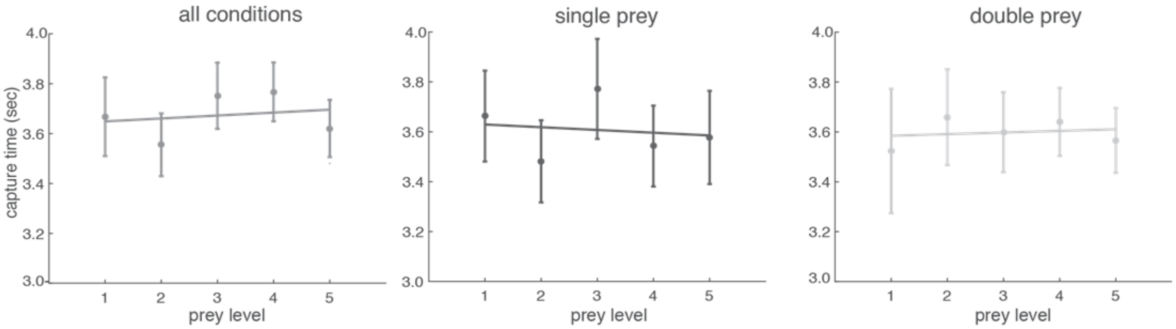

Figure. S1. Flattened capture time regardless of task conditions. We investigated individual conditions for capture time and found that the capture time does not differ among the prey difficulty when it is single prey or double prey. The total number of trials in each subject was 5002 across 19 experimental sessions for subject K and 3094 across five experimental sessions for subject H. To see the main effect according to trial conditions, we pooled all the trials across the subjects.

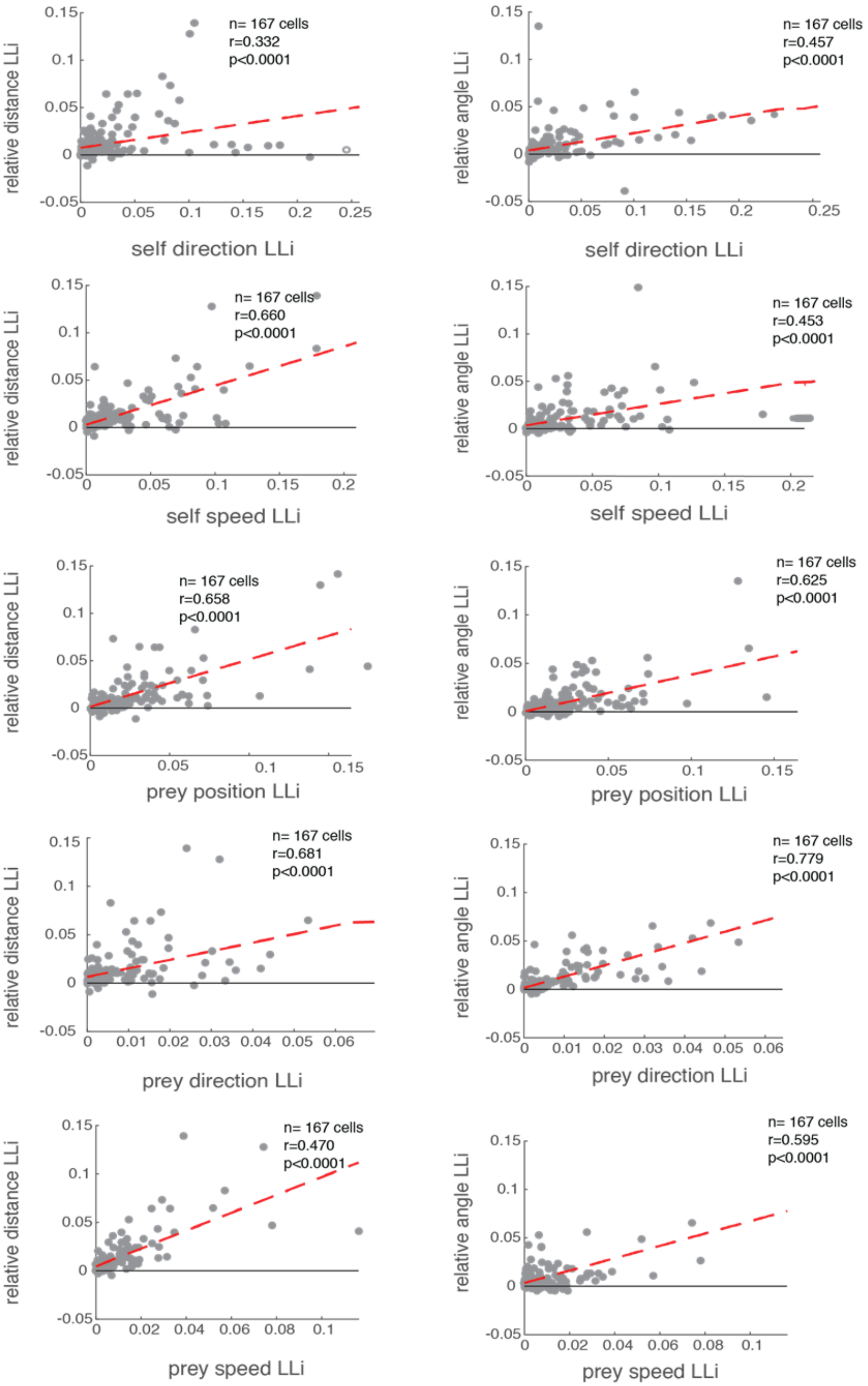

Figure. S2 Log-Likelihood comparison between multiple variables. Scatter plots illustrating correlation between log-likelihood increase (LLi) for various experimental variables. Each dot corresponds to one neuron. Red line indicates the line obtained from linear regression (r-value and p-value is at each panel).

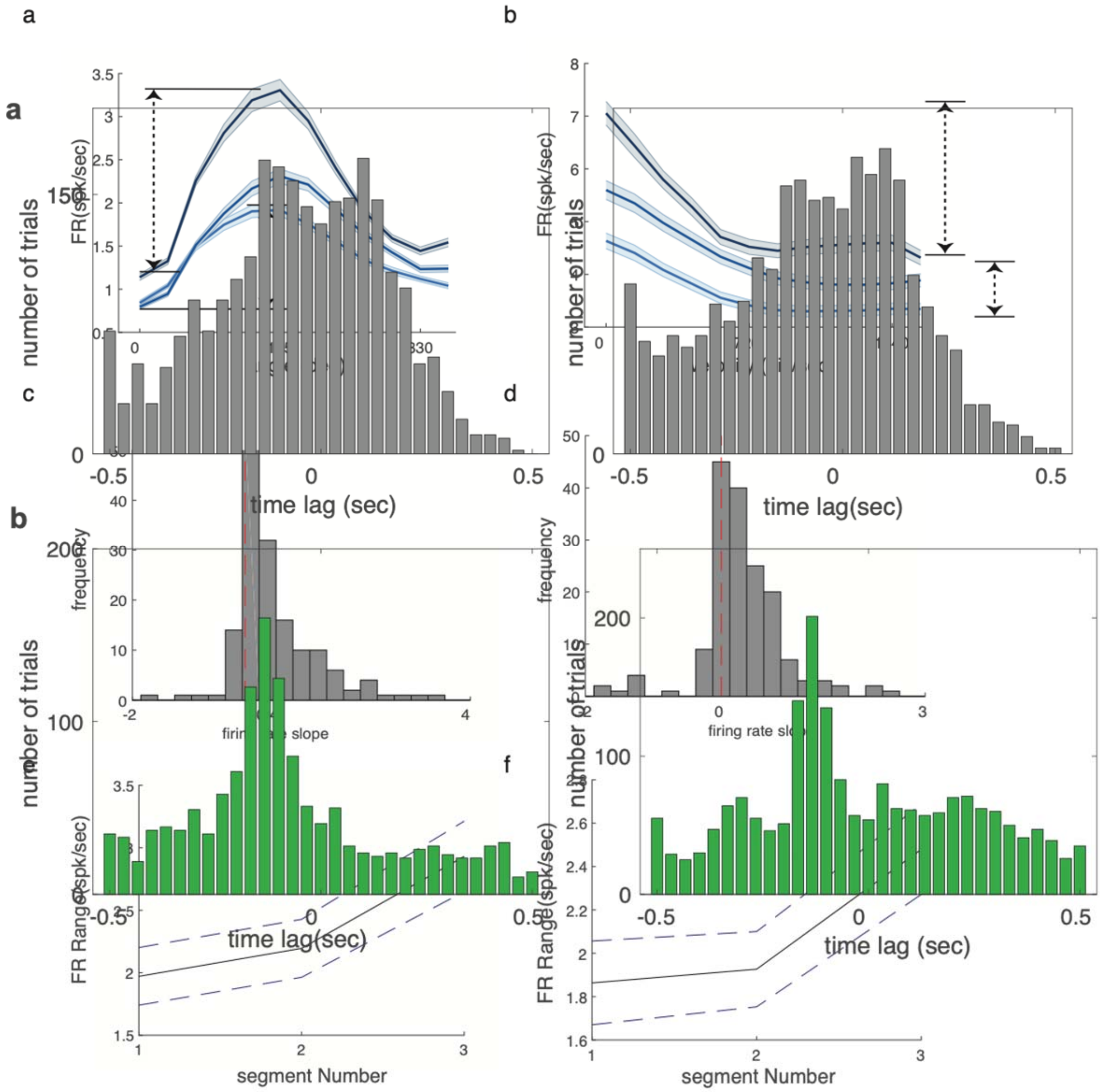

Figure. S3 Additional method for examining mixed-selectivity. (A-B) The individual neurons that has unimodal tuning curve (left) and non-unimodal tuning curve (right). Tuning curve shape variability could make the first method used for mixed-selectivity detection less sensitive (the right one has barely significant MF, 1.0194). Thus, adopting the method used in Hardcastle et al., 2017, we measured the firing rate angle in each condition and compared them. If the slope is positive when each condition is plotted against the range of firing rate, then it is considered mixed-selective. **(C)** The slope of pooled result for position influence to direction, which is significantly different from zero (slope = 0.4798, $p < 0.001$). **(D)** The slope of pooled result for position influence on speed, which is significantly different from zero (slope = 0.3080, $p < 0.001$). **(E-F)** The mean and standard error of the mean for the firing range at each condition. It shows an increasing trend.

Figure S4. The time lead/lag correlation between the complexity of the trajectory (using. curvature of the trajectory as a proxy) and eye-movement magnitude. (A) The relationship between self-trajectory complexity and eye movement magnitude. The right panel is for subject K, and the left panel is for subject P. (B) The relationship between prey trajectory and eye-movement magnitude.

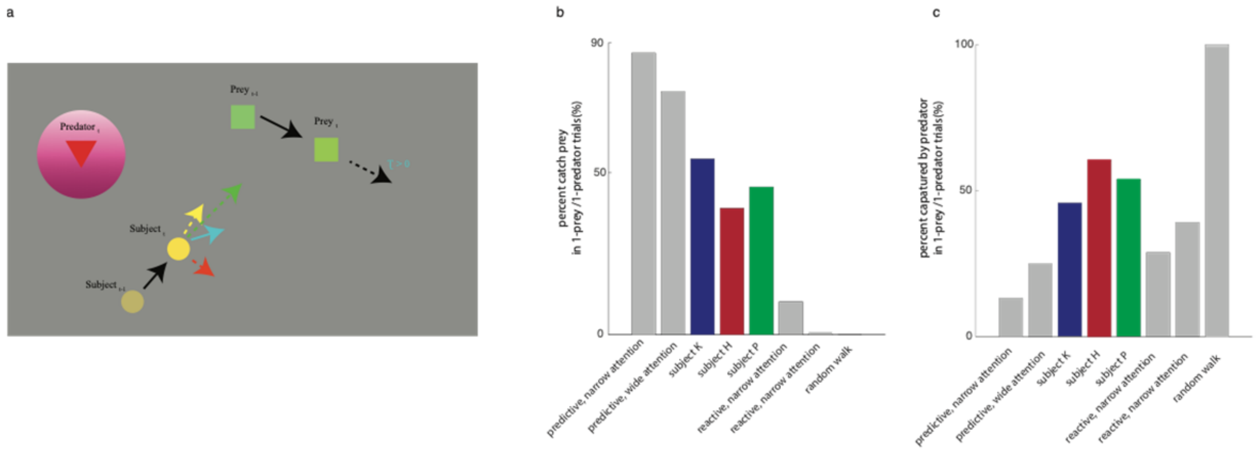

Figure S5. (A) Illustration of the algorithm of a newly simulated agent for estimating the efficiency of the subject's pursuing. The algorithm incorporates selective intake of the predator information depending on the distance (shaded red region) and predictive pursuit components, which is denoted as tau (Yoo et al., 2020). The physical inertia (yellow arrow), pursuit towards prey (cyan arrow), and avoid from a predator (red arrow) are summed and resulted in a single vector (green dashed arrow). (B) Percent of catching prey in 1-prey, 1-predator trials, where inefficient pursuit may end up being captured by a predator. All subjects were between the fully predictive and fully reactive agents with an identical amount of tau parameters. (C) Percent of being captured by the predator within 20 seconds. The result in (B) and (C) may not sum to 1 because there are trials for time outs.

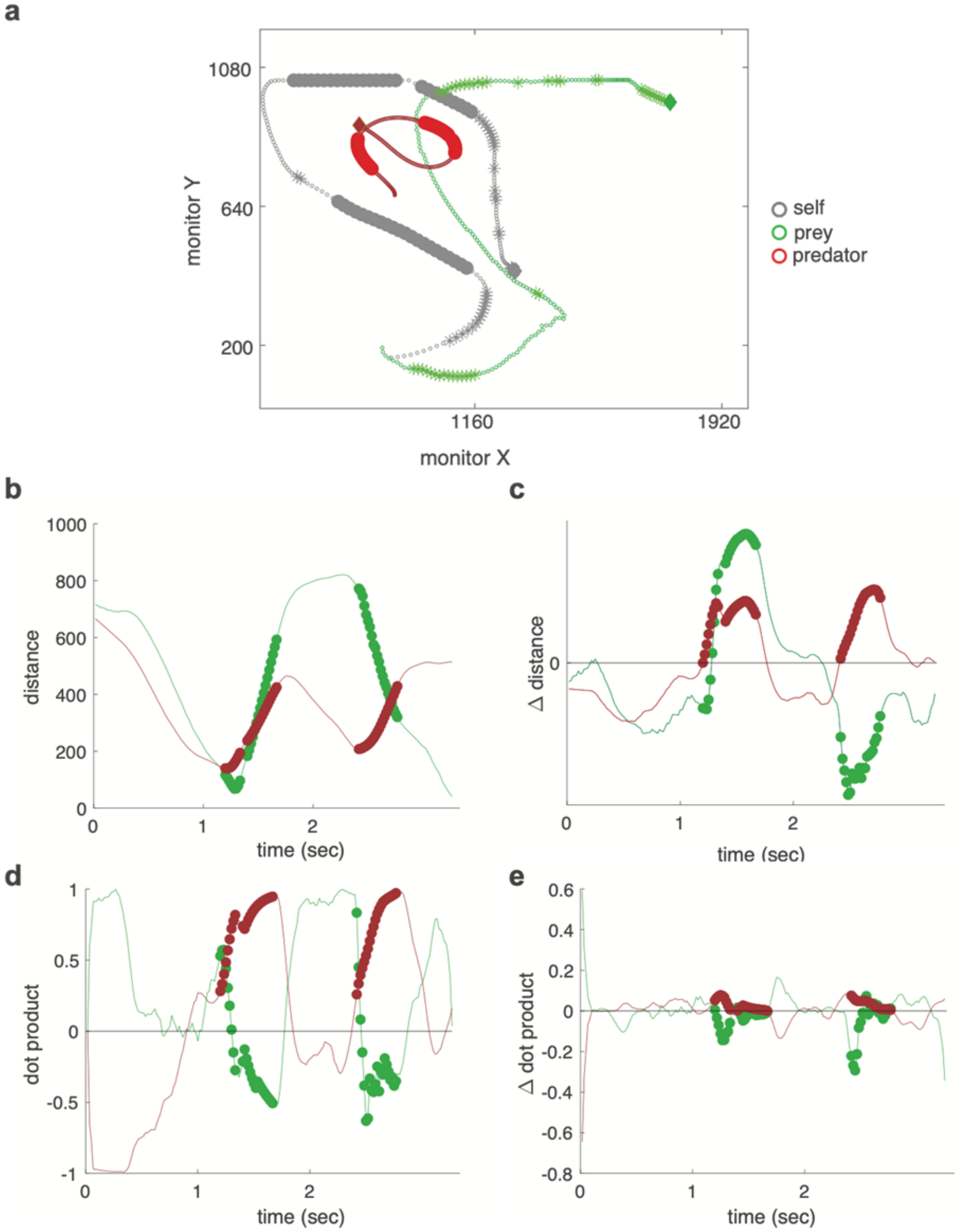

Figure S6. Transition between chase and evade behaviors in the prey pursuit task. Gray: self. Red: predator. Green: prey. (A) Trajectories of self, prey, and predator in an example trial. Red: the distance between self and predator. Green: the distance between self and prey. The filled circles indicate periods of avoidance, as detected by our algorithm. A cross indicates the chase periods, and small unfilled circles indicate periods that are not successfully classified. The filled diamonds points are the location of each agent at the starting of the session. (B) distance between subject-prey and subject-predator over time in an example trial (same trial as in panel A). Filled circles indicate avoidance periods. (C) A derivative of distance as a function of time (same example trial as in panel B). (D) The dot product between the vector of self-movement and other agents is the critical intermediate variable our algorithm uses to classify behavior. A value of -1 indicates that each agent's movements have opposite directions (note the magnitude is converted to 1 as a unit vector), and 1 means the movement directions are aligned. (E) A derivative of the dot product (i.e., of panel D).
