# Supplementary material for: Multicentric tracking of multiple agents by anterior cingulate cortex during pursuit and evasion
Source: Nat Commun. 2021 Mar 31;12:1985. doi: 10.1038/s41467-021-22195-z (PMC8012621; doi:10.1038/s41467-021-22195-z)
Supplement: Supplementary file 3 — Description of Additional Supplementary Files [file 41467_2021_22195_MOESM3_ESM.pdf]

Description of Additional Supplementary Files

File Name: Supplementary Movie 1
Description: The chaired nonhuman primate controlling joystick to perform a dynamic pursuit-avoidance task (left screen on the video). The subject controls a yellow avatar by customized joystick. The goal of the subject is to capture prey (square avatar with distinctive color) and avoid the predator (triangle avatar). The video includes yellow prey, which is removed later to minimize the confusion of color between subject and prey.
